# Acquired bipolar cell disorder presenting with photophobia

**DOI:** 10.1186/s12886-025-04353-9

**Published:** 2025-09-30

**Authors:** Yuro Igawa, Miho Hashimoto, Arisa Yoshida, Satomi Konno, Midori Tachibana, Yozo Miyake, Kei Shinoda

**Affiliations:** 1 Departments of Ophthalmology, Faculty of Medicine, Saitama Medical University, 38 Moro-Hongo Moroyama-machi, Iruma-gun, Saitama, 350-0495 Japan; 2 Department of Ophthalmology, Nagoya University Graduate School of Medicine, Nagoya, Japan

**Keywords:** Acute diffuse occult inner retinopathy, Bipolar cell dysfunction, Negative electroretinogram, Photophobia

## Abstract

**Background:**

Patients have been recently reported who had a common symptom of unilateral photophobia. The electroretinograms (ERGs) recorded from the eyes of these patients had a negative shape with the a-wave normal to slightly attenuated, and the rod and cone responses severely reduced, i.e., a-wave > b-wave. The patients did not complain of night blindness, and the visual acuity and color vision were relatively well preserved. These patients were diagnosed with acute diffuse occult inner retinopathy (ADOIR). We report our findings in such a case in which the unilateral findings progressed to the fellow eye. In the end, we diagnosed our case with bilateral ADOIR.

**Case presentation:**

The patient was a 75-year-old woman whose main complaint was photophobia. She had undergone cataract surgery on both eyes by a neighborhood ophthalmologist. However, her symptoms did not improve, and she was referred to the Saitama Medical University Hospital in May 202X. At our initial examination, she did not complain of night blindness, and her decimal visual acuity in the right eye was 1.0 and that of her left eye was 0.9. The fundus photographs and optical coherence tomographic (OCT) images and Goldmann perimetric visual fields were within normal limits. The full-field mixed electroretinograms (ERGs) had a negative shape with severely reduced rod, cone, and flicker responses in the right eye. Multifocal ERGs showed reduced responses only in the macula of both eyes.

**Conclusions:**

We conclude that our patient had bilateral ADOIR. The ERGs were the key for reaching this diagnosis and would be important for determining the mechanism of photophobia that occurs at the retinal level.

## Background

Cases have been recently reported in Japan of middle-aged and older individuals who have a sudden onset of photophobia and mixed negative electroretinograms (ERGs). The amplitude of the a-wave was normal but the rod, cone, and flicker responses were markedly reduced. Night blindness was a rare complaint in these patients, and their fundi were normal, and their visual acuity, visual fields, and color vision were relatively well preserved. The electrophysiological findings indicated a diffuse bipolar cell dysfunction and no morphological alterations were detected in the imaging studies. These patients were diagnosed with acute diffuse occult inner retinopathy (ADOIR) [1–4].

We report a case with similar features in one eye and then it developed in the fellow eye, i.e., bilateral ADOIR.

### Case presentation

A 75-year-old woman visited a local ophthalmologist in May 202X complaining of photophobia when the right eye was used. She was diagnosed with cataracts and underwent cataract surgery on both eyes in July, but her photophobia did not improve. She was then referred to our hospital in October for further examinations.

At our initial examination, the patient reported that she began to experience glare about a year earlier, and five months earlier she noticed that she had photophobia after several hours of photopsia when using her right eye. Since then, she has noticed a whitish, glowing vision when her right eye was used. She has difficulty walking outdoors, and she wore sunglasses in the summer. She did not complain about night blindness, and there was no significant medical, drug, or family history of ocular disorders.

**Fig. 1:**
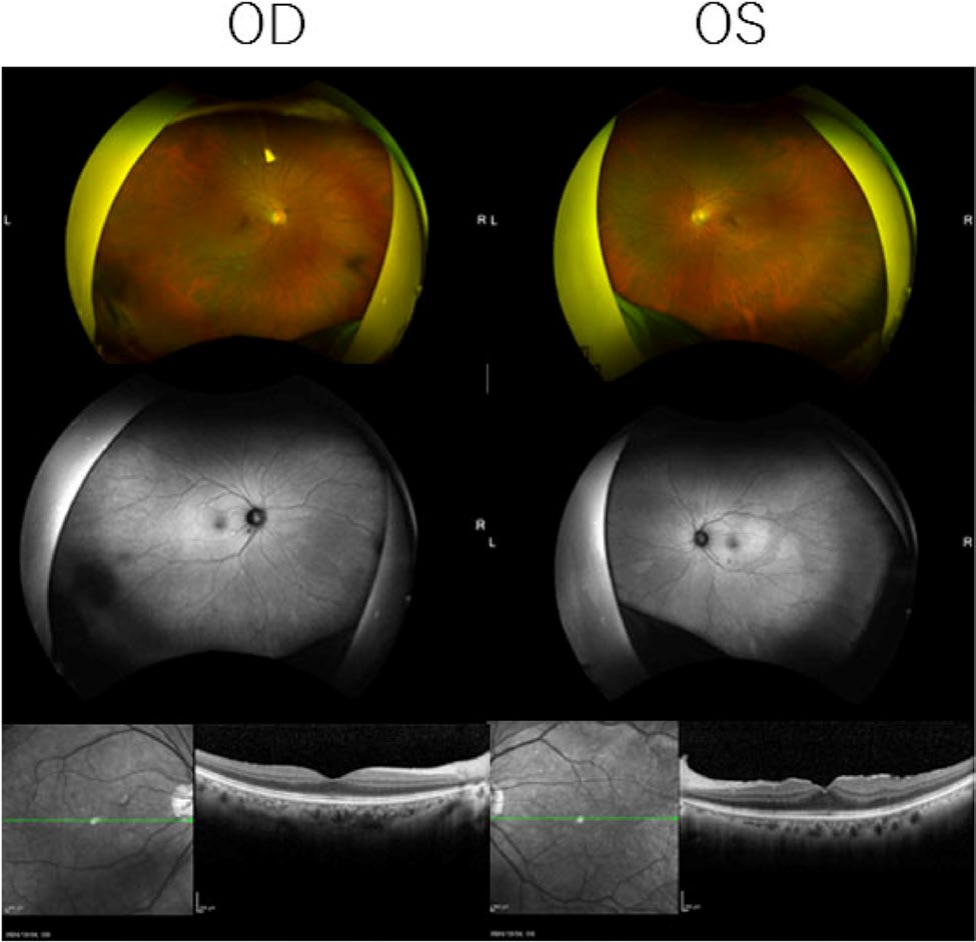
Fundus photograph, fundus autofluorescence image, and optical coherence tomography (OCT) B-scan image Top: No abnormal findings are seen in the fundus photograph Middle: No abnormal findings are seen in the fundus autofluorescence image Bottom: No abnormal findings are seen in the optical coherence tomography (OCT) B-scan image

**Fig. 2:**
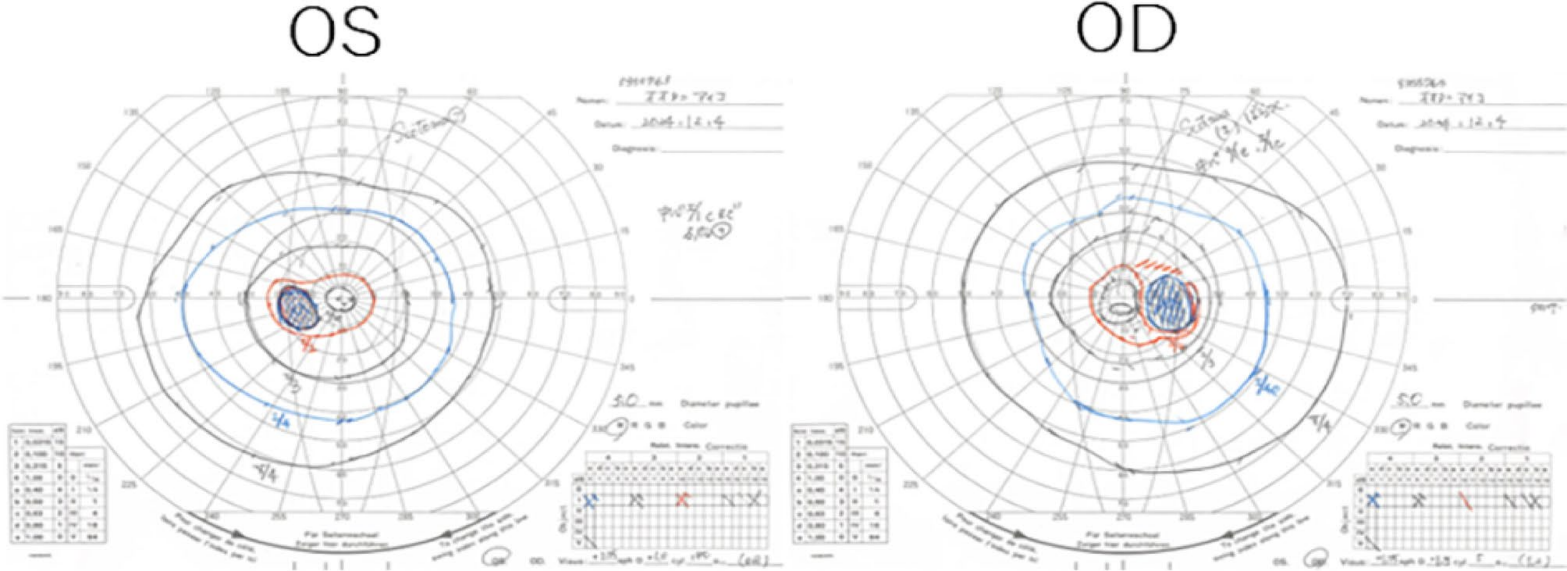
Visual fields determined by Goldmann perimeter Goldmann perimeter was able to measure up to 1/1e in both eyes, and no loss of sensitivity was observed

Our examination found that her decimal best-corrected visual acuity (BCVA) was 1.0 in the right eye and 0.9 in the left eye, and the intraocular pressure (IOP) was 14 mmHg in the right eye and 13 mmHg in the left eye. Slit-lamp examination did not detect any abnormalities in the anterior segment. The ultra-widefield fundus (Optos^®^ California, Nikon Solutions Corporation, Tokyo) images and fundus autofluorescence (FAF) images showed no abnormal findings in both eyes **(**Fig. 1**).** The spectral domain optical coherence tomographic (SD-OCT; Cirrus HD-OCT, Carl Zeiss Meditec) images showed an epiretinal membrane in the left eye **(**Fig. 1**)** but the images were otherwise normal. Goldmann kinetic perimetry found no sensitivity loss in the visual fields **(**Fig. 2**)**.

**Fig. 3:**
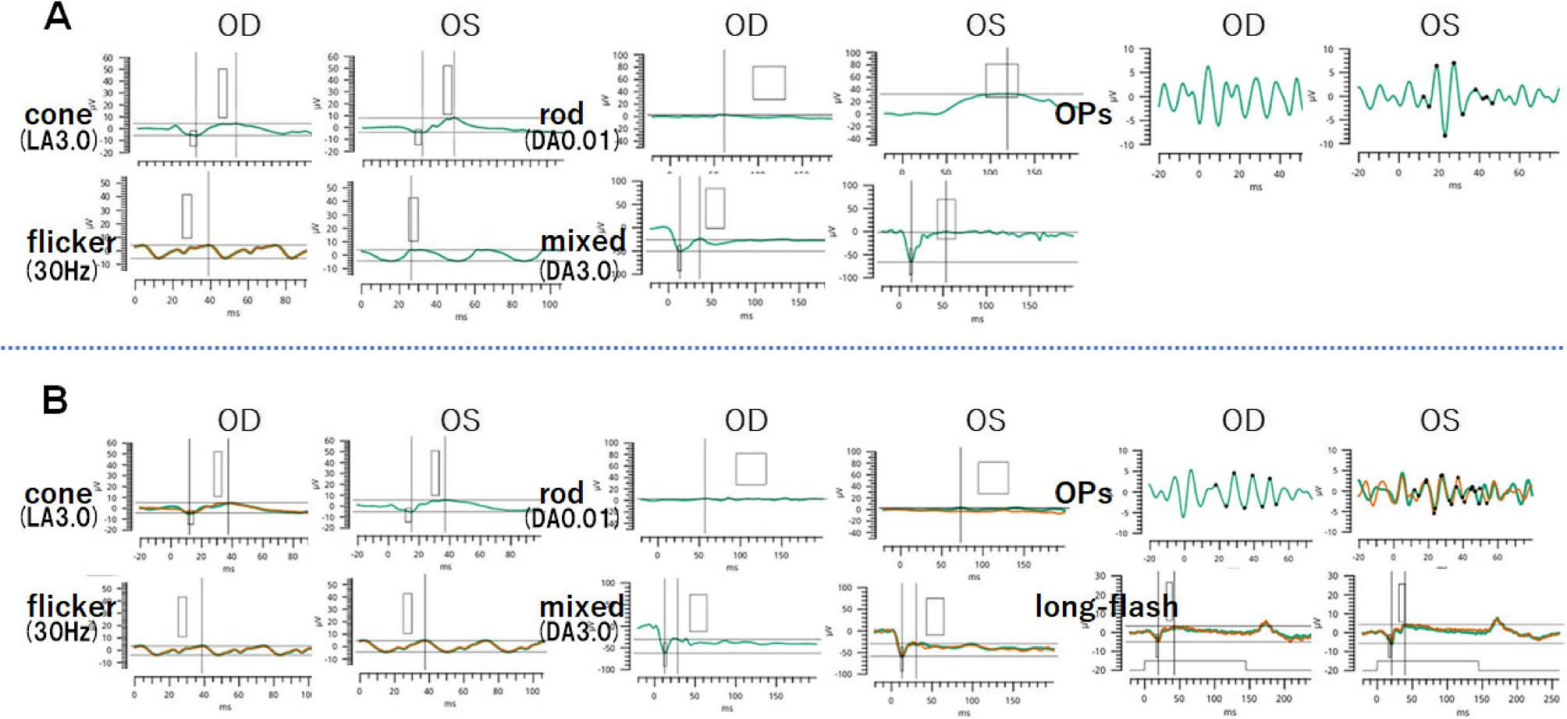
Results of full-field electroretinography recorded at the initial visit (**A**) and 4 months later (**B**) The cone (LA3.0) and flicker responses are attenuated in both eyes. The rod response (DA0.01) is reduced in the right eye. The mixed response (DA3.0) has a negative shape with a marked attenuation of the b-wave in the right eye. The b-wave amplitude is also reduced in the left eye The cone (LA3.0) and flicker responses are further reduced in both eyes. The rod response (DA0.01) is reduced in both eyes, and the mixed response (DA3.0) is negative in both eyes. The on response of the cones following long duration stimuli is severely attenuated and the off-response is moderately reduced in both eyes

**Fig. 4:**
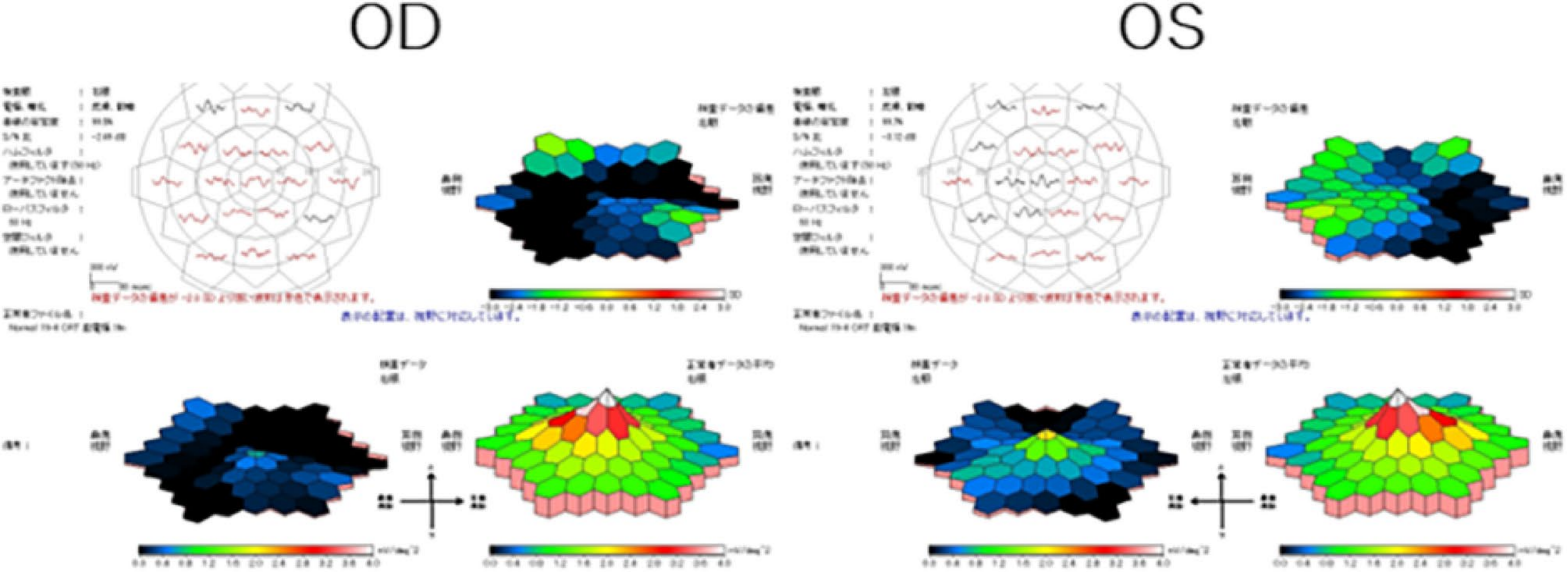
Results of multifocal electroretinograms Multifocal electroretinograms indicated widespread loss of responsivity with responses remaining only in the macular area in both eyes. The left eye is less affected. The response in the macula area of the left eye is better preserved than that of the peripheral responses

Full-field electroretinograms (ffERGs) were recorded according to ISCEV standards [5]. The ERG responses recorded with the RETeval^®^ฏ system (LKC Technologies, Gaithersburg, USA) using skin electrodes in November 202X showed that the flicker responses were significantly reduced in both eyes **(**Fig. 3**)**. Both the a- and b-waves were markedly attenuated in both eyes. The scotopic responses (DA0.01) were almost extinguished in the right eye but preserved in the left eye. The mixed rod-cone ERGs (DA3.0) had a negative shape with a marked reduction of the b-wave in the right eye, and a decrease in the b-wave amplitude in the left eye. The multifocal ERGs (mfERGs, LE-4100, Mayo Co., Ltd., Inazawa) were reduced throughout the visual field except in the macular area of the right eye **(**Fig. 4**)**. The left eye had a relatively good response in the macular area, but there was a general decline in the periphery. These findings indicated a predominant alteration of the macular region of the left eye. In addition, the ffERG recorded 4 months later showed that the flicker responses and cone responses (LA3.0) were further decreased in both eyes **(**Fig. 3**)**. The rod responses (DA0.01) were almost absent in both eyes, and the mixed response (DA3.0) had a negative shape (b-wave < a-wave) in both eyes. The photopic ERGs elicited by long-duration stimuli showed that both the on-response and the off-response were reduced. The color vision was normal in both eyes.

Based on these clinical findings and course, we diagnosed the patient with bilateral ADOIR.

## Discussion and conclusions

Our patient had the symptoms and signs of ADOIR and had ERG finding indicating bipolar cells dysfunction in one eye at the beginning and in the fellow eye 5 months later.

The ERG findings that were key to the diagnosis of ADOIR were the negative ERGs indicating widespread decrease in function of the inner layers of the retina, whereas the mfERGs indicated that the macula was relatively better preserved. Thus, there was only slight impairment of the visual acuity, and the subjective impairment was mainly photophobia with no other complaints.

We recently examined a similar case (Supplemental Figs. 1, 2, Konno, Ganka Rinsho Kiyo, 2025.[in Japanese]). This case shared some features found in the current patient, viz., the patient was elderly, the symptom at the onset was photophobia, the mixed ffERGs responses were negative with the cone and rod responses significantly reduced. The mfERGs showed widespread loss of responsivity with responses remaining only in the macular area in both eyes. The visual acuity was not reduced and there was no complaint of night blindness in that patient.

There are many diseases that present with negatively-shaped mixed ffERGs (Table 1) [1–4, 6–19]. In disorders in which rod photoreceptor function is largely lost, such as vitamin A deficiency, fundus albipunctatus with variants in the *RDH5* gene, or Oguchi disease, the dark-adapted ERGs in a cone isolated retina can have a b-wave of lower amplitude than the a-wave amplitude, a negative ERG according to the photopic hill phenomenon [20]. However, our case was different from those eyes because our case had a significant reduction of the cone responses. Our case can be differentiated from other types of disorders with electronegative ERGs by the clinical course, the fundus and OCT findings, and other test findings.

**Table 1:**
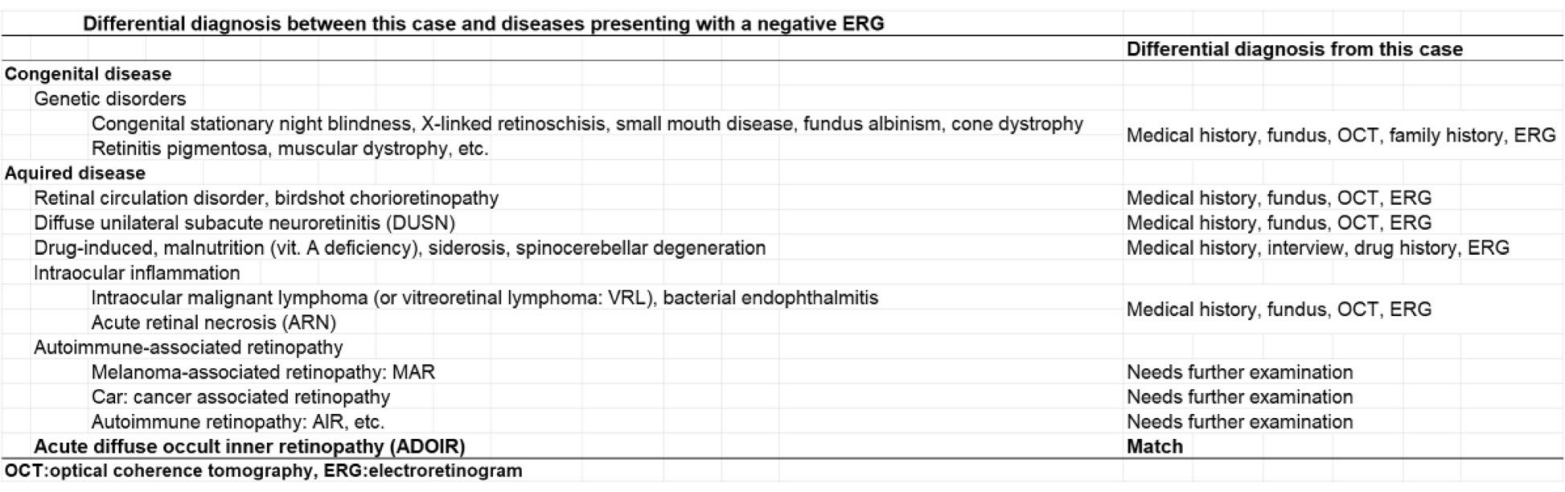
Differential diagnosis between this case and diseases presenting with a negative ERG

However, the possibility of tumor-associated retinopathies such as cancer-associated retinopathy (CAR) [21] and malignant melanoma associated retinopathy (MAR) [22] and autoimmune retinopathy [23] cannot be ruled out. Although the fundus findings and visual field test findings were preserved despite the widespread retinal dysfunction as indicated by the ERG findings, this is unlikely. However, further examinations including systemic examinations, and tests for tumor markers and autoantibodies are required for a definitive diagnosis.

The ffERG findings indicated that the cone and rod responses were significantly reduced or absent, and the mixed responses had a negative shape which suggested a dysfunction of the inner retina. In addition, the responses of the cones to long duration stimuli had an almost complete absence of the on-responses and moderate reduction of the off-responses in both eyes. These findings suggested that both the on- and off-pathways of the bipolar cells were reduced with the on-pathway being more reduced. In addition, the mfERGs of the central field were better preserved. In the two ffERG responses, the right eye was first affected, and changes in the left eye appeared 5 months later. These progressive changes suggested that the ffERG recorded the condition before the progression from unilateral to bilateral ADOIR.

The characteristics of the clinical and laboratory findings in previous reports are shown in Table 2. Our case shared many features. In the previous studies, there were 14 unilateral cases and 3 bilateral cases. In our case, the ERG findings were observed only in the right eye at the onset, but the left eye showed similar findings 5 ​​months later. In our other case whose findings are shown in the Supplemental Figures, the diffuse bipolar cell dysfunction was initially seen in one eye, but after several months, it was detected in the fellow eye, and the condition became bilateral.

**Table 2.**
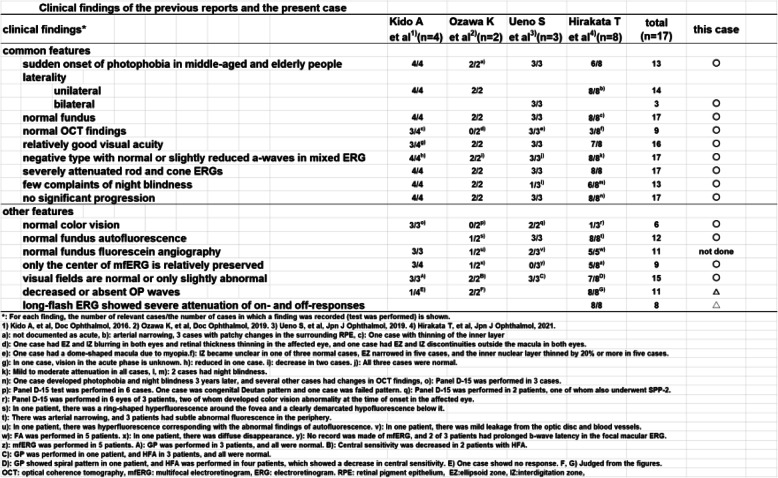
Clinical findings of the previous reports and the present case

In both of our cases and the previously reported cases, fundus photographs, OCT, and visual field results did not provide sufficient evidence for a diagnosis of ADOIR, and only the ERGs detected dysfunction of the inner layer of the retina. This indicated that the ERG findings are very important in diagnosing cases with photophobia as the main complaint.

The causes of photophobia are diverse and include organic diseases and psychogenic diseases. In daily clinical practice, when diagnosing cases with sudden acquired photophobia as the main complaint, if the cause cannot be identified using slit-lamp microscopy, fundus examination, and fundus imaging, ERG recordings should be performed. Our case showed the importance of ERG recordings in determining the mechanism causing the photophobia that occurred at the retinal level.

In previously reported cases, symptoms began suddenly and without warning, even in cases where symptoms had begun more than a year earlier. When we examined the onset alterations and changes in symptoms in the reported cases [1–4], 14 of the 17 cases reported had an acute onset. Similarly, in 8 of these 17 cases for which the follow-up period could be confirmed, the mean duration of symptoms was 6.5 ± 3.3 years (± standard deviation) with a range of 2 to 10 years.

The ERG findings in our patient indicated that this disorder is progressive. Previous reports have reported that the symptoms did not change for years in many cases [1–4]. Thus, the possibility of developing severe visual impairments is low. In only one case was cataract surgery performed for photophobia, but it was not effective [4]. If an autoimmune mechanism is involved, steroids and immunosuppressants may be considered as treatment options, but a determination of the pathology is lacking.

The ERG amplitudes recorded with skin electrodes have been reported to be significantly reduced by approximately two-thirds of those recorded with corneal or DTL electrodes [24, 25]. However, the waveform and implicit time are comparable and do not interfere with identifying the negative type ERGs.

More specifically, full-field ERGs were recorded according to ISCEV standards simultaneously with the ERG-jet corneal contact lens electrodes and the LKC Technologies Sensor Strip adhesive skin electrodes in a recent study [25]. The authors reported that the overall ERG amplitudes obtained with skin electrodes were about one-third of those recorded with contact lens electrodes. The absolute noise levels were comparable, and there was roughly a threefold decrease in the signal-to-noise ratio for ERGs obtained with skin electrodes compared with contact lens electrodes. The authors concluded that it will likely be important to balance the enhanced acceptability of skin electrode protocols for many patients against the reduced statistical power inherent in the noisier ERG recordings they provide.

As to whether the skin electrodes are suitable for clinical use, ERGs are recorded with the RETeval system using skin electrodes not only in children but also in adult cases, especially, when the patient has ocular surface disease or infectious eye disease, and discomfort with corneal electrodes. The use of skin electrodes is practical when repeated examinations are needed.

This study has limitations. Immunoblot analyses for retinal antibodies were not performed, and tumor markers and imaging of the head and body to search for cancer has not been performed. They will be performed after obtaining the patient’s consent. Therefore, we cannot exclude the possibility of diseases such as AIR, CAR, or MAR [15].

In conclusion, our patient presented with photophobia and slit-lamp microscopy, fundus examination, and fundus imaging could not determine the basis for the photophobia. The b-waves of the ERGs were not normal and the ERG findings indicated severe abnormalities in the inner retinal layer function. These findings suggested a pathology similar to ADOIR. The ERGs were the key findings for reaching this diagnosis and should be important for determining the mechanism of photophobia that occurs at the retinal level.

## Supplementary Information

Additional file 1. An 82-year-old woman complained of photophobia without night blindness and her visual acuity was 0.5 in the right eye and 0.6 in the left eye. The fundus, optical coherence tomographic images, and Goldmann and Humphrey visual field tests were normal. Full-field electroretinograms (ERGs) had a negative type ERG with mild attenuation of the a-wave in the mixed rod and cone response, and marked attenuation of the rod response in the right eye (A). Five months later, the ERGs had a negative shape with marked attenuation of the rod response in the left eye (B). The cone response, on-off response, and flicker response were markedly reduced.

Additional file 2. Multifocal electroretinograms recorded from the same patient as Supplemental Fig. 1. These recordings were made 2 months before Supplemental Fig. 1B, The second ffERGs show some residual response only in the macula in the right eye.

## Data Availability

The datasets used and/or analysed during the current study are available from the corresponding author on reasonable request.

## Abbreviations

ADOIR: Acute diffuse occult inner retinopathy
BCVA: Best-corrected visual acuity
CAR: Cancer-associated retinopathy
ERG: Electroretinogram
ffERGs: Full-field electroretinograms
FAF: Fundus autofluorescence
IOP: Intraocular pressure
MAR: Malignant melanoma associated retinopathy
mfERGs: multifocal electroretinograms
SD-OCT: spectral domain optical coherence tomography
